# A pilot study of pre-operative motor dysfunction from gliomas in the region of corticospinal tract: Evaluation with diffusion tensor imaging

**DOI:** 10.1371/journal.pone.0182795

**Published:** 2017-08-22

**Authors:** Bo Gao, Xudong Shen, Mark S. Shiroishi, Mingfan Pang, Zhiqian Li, Benxia Yu, Guiquan Shen

**Affiliations:** 1 Department of Radiology, Yantai Yuhuangding Hospital, Yantai, Shandong, People’s Republic of China; 2 Department of Radiology, Enshi Central Hospital, Enshi, Hubei, People’s Republic of China; 3 Department of Radiology, Keck School of Medicine, University of Southern California, Los Angeles, California, United States of America; 4 Chinese Center for Disease Control and Prevention, Beijing, People’s Republic of China; 5 Department of Radiology, Affiliated Hospital of Guizhou Medical University, Guiyang, Guizhou, People’s Republic of China; Houston Methodist Hospital, UNITED STATES

## Abstract

**Background and purpose:**

Brain tumors in the corticospinal tract (CST) region are more likely to cause motor dysfunction. The aim of this study is to evaluate the effect of gliomas located in the CST region on motor function with diffusion tensor imaging (DTI) preoperatively.

**Materials and methods:**

Forty-five patients with histopathologically confirmed gliomas were included in this pilot study, in all cases (low-grade n = 13, high-grade n = 32) CST but not the motor cortex were involved by the tumor. DTI image were acquired and the posterior limb of the internal capsule fractional anisotropy (FA) and relative FA (rFA = affected FA/contralateral FA) were measured. Injury of the CST from tumor was divided into three grades (grade 1: displacement, grade 2: displacement and infiltration, grade 3: displacement and disruption). The fiber density index (FDi) and relative FDi (rFDi = affected FDi/contralateral FDi) of the injured and contralateral CST were measured. The correlations between muscle strength and the CST injury grade and the rFA, affected FDi, rFDi values were calculated using *Spearman* rank correlation analysis. rFA and rFDi values of muscle strength groups (MMT2-5) were compared with one-way *analysis of variance (ANOVA)*. The difference of muscle strength between low- and high-grade glioma groups were analysed with the *Mann-Whitney U-test*.

**Results:**

Muscle strength was negatively correlated with the injury grade of the CST (*r* = -0.840, *P*<0.001). Muscle strength was positively correlated with rFA, FDi and rFDi (correlation coefficients (r) were 0.615, 0.643 and 0.567 for rFA, FDi and rFDi, respectively). The rFA values between grades (2&3, 2&4, 2&5, 3&5, 4&5) of muscle strength were significantly different (*P*<0.05), the rFDi values between grades (2&4, 2&5, 3&4, 3&5) of muscle strength were significantly different (*P*<0.05), while the rFA and rFDi values in the remaining groups of muscle strength grades showed no significant differences(*P*>0.05).

**Conclusions:**

Preoperative DTI and diffusion tensor tractography may quantify the injury degrees of CST and the extent of motor dysfunction in patients with brain glioma.

## Introduction

Gliomas are the most common malignant tumors of the central nervous system. Brain tumors occurring in the motor cortex may cause reorganization of the motor function area[1]. Voluntary movement is mainly controlled by the primary motor cortex, premotor cortex, and supplementary motor area. Therefore, tumors in these regions may not necessarily cause severe limb dysfunction. The corticospinal tract (CST) passes through the spinal interneuron (Rexed gray matter lamina IV layer), then to the proximal anterior horn motoneuron (control of trunk and limb muscles), or terminate in the spinal cord anterior horn motor cell to dominate free movement of skeletal muscle (control of fine movements of hand, foot and small muscles). Brain tumors located in the CST region are more likely to cause motor system dysfunction. Limb muscle strength is an important index to predict clinical prognosis and to evaluate quality of life in such patients[2].

Compared to conventional MRI, diffusion tensor imaging (DTI) can infer the extent of glioma invasion by evaluating changes of water molecule diffusion in white matter fiber tracts[3–5]. The surgical treatment of intracranial tumors near the CST remains a challenge because of the uncertainty in predicting postoperative limb function assessment is usually more difficult. Using DTI for the quantitative analysis the infiltration degree of the CST has been proven to be critical in the evaluation of motor dysfunction and surgical guidance[6,7].

In this pilot study, we utilize preoperative DTI to study the effect of gliomas located in the region of the CST region on motor function.

## Materials and methods

### Study population

The study was approved by the institutional review board (IRB) of Hubei Enshi Central Hospital, China. All patients provided written informed consent. For minors, this study was carried out following the written informed consent of the parents or guardian.

Fifty-two patients with suspected primary supratentorial gliomas at Hubei Enshi Central Hospital were enrolled in this study from February 2013 to April 2016. Tumors were diagnosed with pre-operative conventional and contrast-enhanced MRI and were classified by two neuroradiologists with more than five years interpretation experience, according to World Health Organization 2007 criteria[8] as confirmed by surgery and biopsy. All histopathological data were provided and reviewed by department of pathology of Hubei Enshi Central Hospital and Guizhou Medical University.

The inclusion criteria were the followings: 1) histopathologically confirmed glioma; 2) MR imaging examination prior to surgery; 3) no radiation or chemo-therapy before the MR imaging examination; 4) tumors located in the cerebral hemispheres in the region of the ipsilateral CST; 5) contralateral CST demonstrating normal appearance or only mild displacement on FA color-coded map. The exclusion criteria were the followings: 1) evidence of tumor located in the primary motor cortex or centered in this region demonstrated on contrast-enhanced MR images or abnormal T2-hyperintensity; 2) CST regions affected by other diseases such as leukodystrophy, vascular diseases, etc; 3) lesions in the motor cortex may lead to limb motor dysfunction such as cerebral infarction, infection,etc; 4) obvious motion artifacts; 5) muscle motor dysfunction caused by other diseases such as limb disuse, arthrogenous, osteoporotic or joint soft tissue injury.

Contralateral knee muscle strength of all patients was measured by manual muscle testing (MMT). The knee muscle strength was divided into 0–5 grade (0: no evidence of muscle contraction, 1: contraction but no movement, 2: movement with without gravity, 3: movement against gravity but not against resistance, 4: movement against resistance but less than normal, and 5: normal strength). The test was repeated three times and the muscle strength grade was determined by a neurologist with more than five-year experience and who are blinded to imaging results.

### MR imaging

MRI scans were performed on a Philips Achieva X-Series 3.0T system with a 16-channel SENSE head coil. All patients underwent conventional contrast-enhanced brain MRI and DTI scans. Routine MRI scanning protocols include axial T1-weighted imaging (T1WI), axial and sagittal T2-weighted imaging (T2WI), and axial T2-weighted fluid-attenuated inversion recovery (FLAIR). T1WI: inversion time, 800 ms; repetition time/echo time, 2278/20 ms; section thickness, 6 mm; matrix size, 288×190; field of view, 196×196 mm. Axial and sagittal T2WI: repetition time/echo time, 2500/90 ms; section thickness, 6 mm; matrix size, 420×306; field of view, 230×230 mm. Axial FLAIR: repetition time/echo time, 8000/120 ms; section thickness, 6 mm; matrix size, 304×216; field of view, 230×230 mm. Axial contrast-enhanced SE T1WI scan: repetition time /echo time 400/8.6 ms; section thickness, 6 mm; matrix size, 288×192; field of view, 230×230 mm. For contrast-enhanced T1WI, 0.1mmol/kg dose of gadolinium-DTPA contrast agent (Bayer Schering Pharma, Magnevist) was administered intravenously. Spoiled gradient echo (FFE) T1WI: repetition time/echo time, 200/2 ms; section thickness, 1 mm; interval 1 mm; flip angle 75°; matrix 256×256; field of view, 230×230 mm. For DTI, diffusion-weighted respiratory-triggered single-shot echo-planar imaging (SS-EPI) was used with the following parameters: repetition time /echo time, 7103.62/60 ms; matrix size, 92×92; field of view, 222×222 mm; 60 sections, 2 mm slices no gap; NEX 2. Diffusion-gradient encoding in 15 directions with *b* value of 800 sec/mm^2^ and an additional measurement without diffusion-gradient encoding (*b* = 0 sec/mm^2^) were performed. The total scanning time was about 50 minutes for each patient.

### ROI measurements

The MR Workspace (Extended V2.6.3.4, Philips Medical System) software package was used for post-processing of DTI and DTT. Data acquisition noise was eliminated with Gaussian smoothing filter. A region of interest (ROI) area measuring about 50 mm^2^ was placed in the posterior three quarters of the posterior limb of the internal capsule of the affected side by two experienced neuroradiologists who had at least five years of work experience and were familiar with the utility of the post-processing methods. FA values were measured by the same two independent readers on the axial FA color-coded map of the healthy and injured side. ROI measurement of the healthy and the injured side were repeated three times and an average value was recorded. This value was used to calculate relative FA values (rFA = injured FA/contralateral FA).

Seed regions were selected using the axial color encoding direction map for CST fiber tracking. The CST originates in the motor cortex (Broadman 4 and Broadman 6), and then extends into the corona radiata, posterior limb of internal capsule, cerebral peduncle, and pyramids of medulla oblongata. Therefore, the ROIs of the CST were placed in the following three areas: pyramids of medulla oblongata, cerebral peduncle, subcortical white matter of precentral gyrus and premotor cortex.

A line propagation technique was used to track fibers and the logic algorithm "AND" was used to include the three ROIs through the fiber tracts, as well as "NOT" to exclude fiber tracts deviating from normal anatomy, using the contralateral corresponding region as a reference. The FA threshold was set to 0.2, the angle of fiber tracing was 27 degrees, and step size was 0.2. FDi is the number of white matter fibers per unit volume that is calculated after the fiber tracing is completed (rFDi = injured FDi/contralateral FDi). Ipsilateral and contralateral CST FDi and rFDi were obtained.

**Fig 1:**
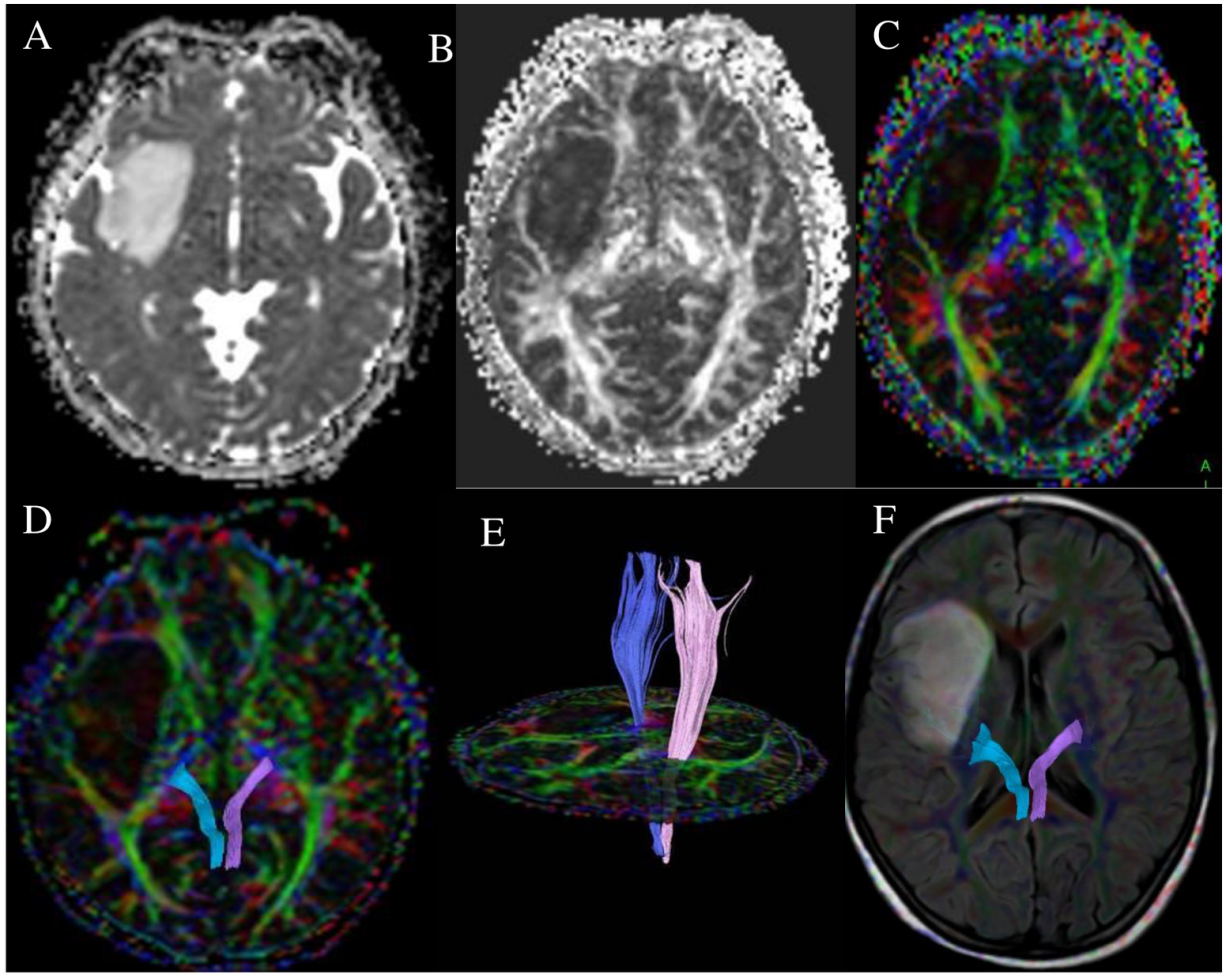
A 40-year-old female with right frontotemporal astrocytoma (WHO grade II) and left lower limb muscle strength was MMT 5. ADC map (A) show the solid part of tumor was slightly lower signal, FA map (B) and color encoding pattern map (C) and DTT map (D, E) on the right side of the corticospinal tract showed mild displacement, DTT-FLAIR show around of the CST without edema.

**Fig 2:**
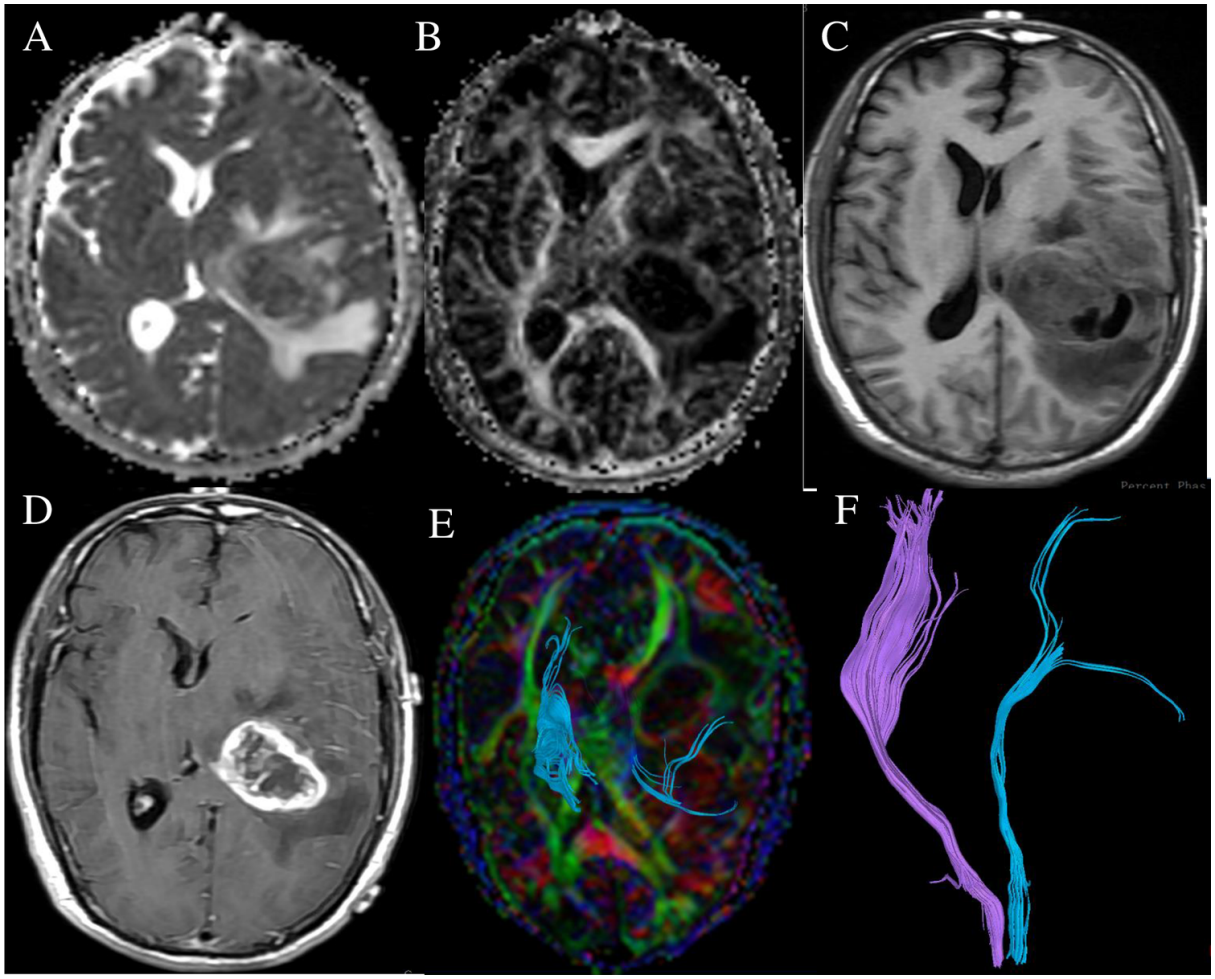
A 48-year-old female with left thalamic glioblastoma multiforme (WHO grade IV), the right knee muscle strength was MMT 3. ADC map (A), FA map (B),T1WI(C), contrast-enhanced T1WI (D) show the tumor infiltrated normal tissues. DTT map (E, F) show the left corticospinal tract was displaced and infiltrated, and the number of left corticospinal fibers decreased.

Witwer et al.[9] and Field et al.’s[10] classification method was employed to quantify the status of the CST relative to tumors. Because nearly all patients in this study had displacement of the CST to a certain extent, infiltration and disruption were defined as follows: 1) grade 1: displacement—white matter tracts display normal signal normal on the FA map or FA color-coded map; 2) grade 2: displacement and infiltration—displacement and abnormal signal on the FA map or FA color-coded map with decreased FA values; white matter fiber tracts surrounded by tissue edema but remained intact without significant disruption (Fig 1); 3) grade 3: displacement and disruption—coexistence of white matter fiber tract displacement and disruption; the direction of white matter tracts cannot be identified on the FA map or FA color-coded map, and interruption of white matter tracts can be found on DTT (Fig 2).

## Statistical analysis

All statistical analysis was performed using SPSS 13.0 (SPSS, Inc., Chicago, IL, USA). The relationship between the injury degrees of the CST, rFA, FDi, rFDi values and muscle strength were analyzed with *Spearman* rank correlation analysis. The rFA and rFDi values of each muscle strength group were tested for normality as well as equality of variances. If the data met the assumption of normal distribution and homogeneity of variance, an independent sample-*t* test was applied. If the data followed a normal distribution and heterogeneity of variance, the approximated independent sample-*t* test was used for analysis. The differences between the mean rFA and rFDi values of different muscle strength groups were analyzed with analysis of variance (*ANOVA*). The difference of muscle strength between low- and high- grade gliomas groups were analysed with the *Mann-Whitney U-test*. Statistical significance was designated as *P < 0*.*05*.

## Results

Of our 52 patients, 4 cases with partial infiltration of the primary motor cortex and 3 cases with very large tumors that precluded fiber tracking were excluded. Therefore, we ultimately enrolled 45 cases that satisfied our inclusion criteria. The average age was 45 (range 6–78) years. Twenty-six were male and 19 were female. Thirty-eight cases demonstrated motor dysfunction. Seven cases exhibited headache, dizziness, convulsions and slight movement dysfunction.

Patient demographics and the results of CST injury grade are listed in S1 Table. CST injury grade: Twenty-one cases demonstrated grade 1, 6 cases showed grade 2, 18 cases had grade 3. Muscle strength: Six cases had grade 2, 12 cases demonstrated grade 3, 21 cases showed grade 4, and 6 cases had grade 5.A negative correlation was found between muscle strength and CST injury grade (r = -0.840, *P*< 0.05) while a positive correlation was found between muscle strength and rFA value of the posterior limb of internal capsule between the injured and contralateral side (r = 0.615, *P*< 0.05). Positive correlations were also noted between muscle strength and rFDi (r = 0.567, *P*< 0.05) and FDi of the injured side (r = 0.643, *P*< 0.05). There were 13 cases with low-grade gliomas and 32 cases with high-grade gliomas, a statistically significant difference in muscle strength was found between these 2 groups of tumors (Z = -4.324, *P*<0.05).

The values of rFA and rFDi between different muscle strength groups were compared. The rFA values between grade (2 & 3, 2 & 4, 2 & 5, 3 & 5, 4 & 5) groups of muscle strength were significant difference (*P*<0.05), the rFDi values between grade (2& 4, 2 & 5, 3 & 4, 3 & 5)groups of muscle strength were significantly difference (*P*<0.05), the rFA and rFDi values in the remaining groups of muscle strength grades had no significant difference(*P*>0.05) in S2 Table.

## Discussion

Tumors in the motor cortex may not result in serious limb dysfunction because of motor function remodeling[11,12]. The purpose of this study was to quantitatively analyze the degree of damage to the conductive fibers and resulting muscle strength. Therefore, we exclude lesions in the motor cortex and lower limb lesions. The statistical results showed that muscle strength was negatively correlated with the degree of CST injury, while a positive correlation was seen with rFA, FDi and rFDi values. Tumor cell proliferation and invasion would result in disruption of CST myelin and axonal membranes, the degree of which may reflect its biological behavior[13,14]. DTI characterizes the movement of water molecules, which can be used to evaluate the proliferation and invasion, as well as Na^+^-K^+^ pump damage of the cell membrane[15]. Nerve fiber tract damage results in the inability of nerve impulses to spread sequentially, thus resulting in reduce of limb resistive exercise. Damage to nerve fiber tracts can lead to a reduction of anisotropic diffusion of water molecules and an increase in isotropic diffusion, with resulting decrease of FA and rFA values[5]. Fiber disruption and tumor infiltration may lead to decreasing units within the fiber voxel with consequent reduction of FDi and rFDi[4]. Consequently tumor infiltrating the CST can lead to CST damage and subsequent motor dysfunction.

DTI parameters may potentially quantify the degree of CST injury, assess the damage to motor conduction fibers preoperatively, and predict therapeutic effect and outcome[16,17,18]. Our statistical results showed that the values of rFA between different muscle strength groups have significant differences. Beppu et al.[19] measured FA values in glioblastoma and normal brain tissue and found that FA may reflect glioma cell density and proliferative activity. Some studies have suggested that FA value is closely related to the integrity of white matter fiber tracts[20,21]. Stadlbauer et al.[22] studied 10 glioma patients with preoperative DTI positron emission tomography (PET) and found that the pyramidal tract rFA value in the sensorimotor deficits group was significantly reduced while ^18^F-FDG uptake was significantly increased, the increase of ^18^F-FDG uptake may be associated with tumor cell proliferation and infiltration. Therefore, changes of muscle strength due to CST injury may be the result of tumor cell proliferation and infiltration degree.

The comparison between different grades of gliomas showed that muscle strength was significantly different between high- and low-grade groups. FA values may reflect the degree of tumor infiltration[3]. Prior studies have found that FA values were reduced in the CST which may be associated with decrease muscle strength in the contralateral limb[23–26]. Different degrees of glioma infiltration may have resulted in inconsistencies in the nerve fiber axon gap and the number of axons in each voxel. The fiber density and anisotropy had no significant changes or just mild changes in our low-grade glioma patients (S1 Table), possibly because the myelin membrane has no obvious or only mild injury in low-grade gliomas. High-grade glioma grow aggressively and so the necrotic microcapsules within the tumor may lead to damage of the surrounding glial cells and neurons with reduction of fiber density[27]. The decrease of the fibers density may then lead to a reduction of brain nerve impulse conduction from the motor cortex resulting in involuntary limb movements. Stadlbauer et al.[18] found that the FDi value of the affected side was significantly lower than that of contralateral side and also that there was a difference of FDi values between high-grade and low-grade groups. They found that both the N-acetyl-aspartate (NAA) peak from MR spectroscopy (MRS) and FDi decrease in high-grade glioma indicated the damage to the integrity of the nerve structure and fibers. Their results suggest that the FDi value can reflect the degree of tumor infiltration better than the FA. FDi was significantly linearly correlated with the number of tumor cells[28]. Muscle dysfunction and the degree of CST injury from glioma were linearly correlated with fiber density suggesting that DTI can reflect different degrees of injury from glioma in CST area.

The action potential conduction in the motor area is influenced by the myelin sheath integrity and diameter of fibers [29]. Theoretically, the closer a brain tumor is to the CST, the greater the potential for decreases of muscle strength. Furthermore, larger tumors may result in higher degree of injury on CST. This may result in smaller axonal diameter and thinner myelin which may cause decreased transmission of nerve impulses[29]. Although prior studies have shown that the size of benign brain tumors was not significantly different between hemiplegia versus non-hemiplegic groups[30], these results may be due to tumor location, duration of diseases, pathological type and sample size among other factors.

Several limitations of our work should be considered. Linear extension technology tracing of fiber tracts is defined based on the diffusion property of water molecules in each voxel, not true fiber tracts[31]. Although a large number of studies have shown a high degree of consistency using fiber tracing techniques with classical anatomy, our results were not confirmed by histopathology. Therefore, our finding of abnormal white matter fibers need to be further confirmed in subsequent studies using histopathological confirmation. Although ROI-based measurement is the most common DTI analysis method, our use of this technique did not adequately consider tumor heterogeneity[32]. With the growth of tumor, microvascular necrosis within the tumor may lead to damage to the surrounding glial cells and neurons; therefore, the pathological concept of "tumor invasion margin" should be introduced into a future study[33].Placement of the borders of the ROI along the edge of the tumor outline is the most commonly used technique[34]. It has been noted that the junction of tumor and normal tissue are rich in cancer stem cells, which might represent tumor invasion, within the range of three to five layers of cells[33]. Our study also found that tumor infiltrating margin was associated with the CST injure grades. Based on the above, we chose the corticospinal tract ROI area to avoid the outline of tumor edge. Our study used only DTI for preoperative assessment of the degree of CST injury. Changes in the dispersion of the fibers caused by tumor infiltration or edema can also potentially be identified by MRS analysis[35]. Blood oxygen level-dependent (BOLD) functional MRI (fMRI) can also localize brain functional areas and display its activation level[36]. Combining MRS, fMRI with DTI may provide more information on the assessment brain tumors and motor function changes[37]. Furthermore, our study provided only preoperative muscle strength grading for patients. Intraoperative cortical or subcortical stimulation were not combined to obtain EEG and postoperative motor function and life quality were not evaluated either. Only the pre-operative motor function of patients was assessed in this study. In addition, language dysfunction was also not assessed here. In our study, only contralateral knee muscle strength was tested. Tumor involving any portion of the CST may lead to lower limb muscles and trunk muscles weakness and so it may be more meaningful to incorporate these muscle strength changes into study in the future. The change of muscle strength is not likely to be the result of one single factor, actually which is affected by primary motor cortex, degree of CST injury and joint muscle disease. Therefore, the clinical evaluation of muscle strength must take these factors into consideration.

## Conclusions

In summary, our study suggests that pre-operative DTI quantitative parameters can assess the degree of CST injury by glioma and show abnormal brain tumor infiltration area. The degree of muscle strength dysfunction is correlated well with the injury degree of the CST. DTI may provide critical information for the greatest degree of tumor resection and protection of conductive fibers.

## Supporting information

S1 Fig A 40-year-old female with right frontotemporal astrocytoma (WHO grade II) and left lower limb muscle strength was MMT 5. ADC map (A) show the solid part of tumor was slightly lower signal, FA map (B) and color encoding pattern map (C) and DTT map (D, E) on the right side of the corticospinal tract showed mild displacement, DTT-FLAIR show around of the CST without edema.
(RAR)

S2 Fig A 48-year-old female with left thalamic glioblastoma multiforme (WHO grade IV), the right knee muscle strength was MMT 3. ADC map (A), FA map (B),T1WI(C), contrast-enhanced T1WI (D) show the tumor infiltrated normal tissues. DTT map (E,F) show the left corticospinal tract was displaced and infiltrated, and the number of left corticospinal fibers decreased.
(RAR)

S1 Table Clinical characteristics of 45 patients with glioma. (DOCX)

S2 Table The comparison of rFDi and rFA among different muscle groups. Note: rFA was the injuryed/contralateral FA value of injured and contralateral side in posterior limb of internal capsule, rFDi was the injuryed/contralateral FDi value of injured and contralateral CST, test level to P <0.05 was considered statistically significant, * indicated statistically significant differences.
(DOCX)

S1 File STROBE_checklist_v4_combined_PlosMedicine. (DOCX)

S2 File ICMJE conflicts of interest-Bo Gao. (PDF)

## Abbreviations

CST: corticospinal tract
DTI: diffusion tensor imaging
DTT: diffusion tensor tractography
FA: fractional anisotropy
FDi: fiber density index
