# Supplementary material for: A pilot study of pre-operative motor dysfunction from gliomas in the region of corticospinal tract: Evaluation with diffusion tensor imaging
Source: PLoS One. 2017 Aug 22;12(8):e0182795. doi: 10.1371/journal.pone.0182795 (PMC5568729; doi:10.1371/journal.pone.0182795)
Supplement: S1 Table — (DOCX) [file pone.0182795.s003.docx]

**Table 1. Clinical Characteristics of 45 Glioma Patients**

| Case number | Sex | Age | Histopathologically and WHO grade | Clinical manifestations | Location of tumor and relative to CST | MMT staging | CST injury  grade |
| --- | --- | --- | --- | --- | --- | --- | --- |
| 1 | M | 55 | Anaplastic astrocytoma(WHO Ⅲ) | Dizziness three months, climbing repeatedly fall | Left frontal and temporal junction areas, CST was compressed and disrupted | MMT 2 | grade 3 |
| 2 | F | 68 | Glioblastoma(WHO Ⅳ) | Dizziness five days with unsteady gait | Right inferior fontal gyrus, CST was compressed and disrupted | MMT 2 | grade 3 |
| 3 | F | 56 | Glioblastoma(WHO Ⅳ) | Left limb weakness, unable to stand, intermittent headache | Right temporal lobe, CST was compressed and disrupted | MMT 2 | grade 3 |
| 4 | M | 61 | Glioblastoma(WHO Ⅳ) | Limb weakness six months | Left corona radiate, CST was compressed and disrupted | MMT 2 | grade 3 |
| 5 | M | 14 | Anaplastic astrocytoma(WHO Ⅲ) | Limb weakness a year | Left basal ganglia, CST was compressed and disrupted | MMT 2 | grade 3 |
| 6 | M | 70 | Glioblastoma(WHO Ⅳ) | Limb weakness， Dizziness | Left frontal and temporal junction areas, CST was compressed and disrupted | MMT 2 | grade 3 |
| 7 | M | 71 | Anaplastic astrocytoma(WHO Ⅲ) | Left limb numbness gradually increased by 4 months | Right temporal lobes, CST was compressed and disrupted | MMT 3 | grade 3 |
| 8 | F | 68 | Glioblastoma(WHO Ⅳ) | Headache for 2 months, increased by 10 days | Right temporal lobes, CST was compressed and disrupted | MMT 3 | grade 3 |
| 9 | F | 52 | Anaplastic astrocytoma(WHO Ⅲ) | Dizziness headache 1 months with unstable walking for 1 days | Right parietal lobe,CST was compressed and disrupted | MMT 3 | grade 3 |
| 10 | M | 61 | Anaplastic astrocytoma(WHO Ⅲ) | Unsteady gait 7 days | Right frontal and temporal junction areas,CST was compressed and disrupted | MMT 3 | grade 3 |
| 11 | F | 62 | Gliosarcoma(WHO Ⅳ) | Sudden dizziness with unstable walking for 1 months | Right temporal lobes, CST was compressed and disrupted | MMT 3 | grade 3 |
| 12 | M | 60 | Glioblastoma(WHO Ⅳ) | Dizziness with unstable walking for half a month | Left middle frontal gyrus, CST was compressed and disrupted | MMT 3 | grade 3 |
| 13 | F | 68 | Glioblastoma(WHO Ⅳ) | Slow speech with right limb weakness activities for about 10 days | Left cingulate gyrus, CST was compressed and disrupted | MMT 3 | grade 3 |
| 14 | F | 46 | Anaplastic astrocytoma(WHO Ⅲ) | Limb weakness for two weeks | Right middle frontal gyrus, CST was compressed and disrupted | MMT 3 | grade 3 |
| 15 | M | 27 | Anaplastic astrocytoma(WHO Ⅲ) | Suddenly collapsed | Multiple astrocytomas in the right hemisphere,CST was compressed and disrupted | MMT 3 | grade 3 |
| 16 | M | 40 | Anaplastic astrocytoma(WHO Ⅲ) | Right limb weakness about a month | Left temporal lobe, CST was compressed and disrupted | MMT 3 | grade 3 |
| 17 | F | 57 | Anaplastic astrocytoma(WHO Ⅲ) | Episodic memory impairment for 6 months | Right temporal lobe, CST was compressed and disrupted | MMT 3 | grade 3 |
| 18 | M | 78 | Glioblastoma(WHO Ⅳ) | Left limb weakness for 24 days | Right temporal lobe, CST was compressed and disrupted | MMT 3 | grade 3 |
| 19 | M | 46 | Astrocytoma(WHOⅠ) | Sudden double lower limbs twitch for 45 days | Right temporal lobe, CST was compressed | MMT 4 | grade 1 |
| 20 | M | 26 | Pilocytic astrocytoma(WHOⅠ) | Transient disturbance of consciousness with twitching limbs one week | Right hippocampal gyrus, CST was compressed | MMT 4 | grade 1 |
| 21 | M | 42 | Oligodendroglioma(WHO Ⅱ) | Sudden convulsions for 17 days, headache and left limb fatigue 5 days | Right temporal lobe, CST was compressed | MMT 4 | grade 1 |
| 22 | M | 46 | Astrocytoma(WHOⅠ) | Paroxysmal convulsions seven years, increased 3 days | Right inferior fontal gyrus, CST was compressed | MMT 4 | grade 1 |
| 23 | M | 37 | Oligodendroglioma(WHO Ⅱ) | Repeated convulsions 8 years | Right inferior fontal gyrus, CST was compressed | MMT 4 | grade 1 |
| 24 | F | 45 | Oligodendroglioma(WHO Ⅱ) | Headache 3 weeks | Left inferior fontal gyrus, CST was compressed | MMT 4 | grade 1 |
| 25 | F | 6 | Pilocytic astrocytoma(WHOⅠ) | Limb weakness | Left thalamus, CST was compressed | MMT 4 | grade 1 |
| 26 | F | 51 | Oligodendroglioma(WHO Ⅱ) | Limb weakness | Left frontal and temporal junction areas, CST was compressed | MMT 4 | grade 1 |
| 27 | M | 45 | Diffuse astrocytoma(WHO Ⅱ) | Headache with nausea for half a month | Right inferior fontal gyrus, CST was compressed and infiltrated | MMT 4 | grade 2 |
| 28 | F | 48 | Anaplastic astrocytoma(WHO Ⅲ) | Sudden fainting with coma, dizziness 8 hours | Left temporal lobes, CST was compressed and infiltrated | MMT 4 | grade 2 |
| 29 | M | 36 | Anaplastic astrocytoma(WHO Ⅲ) | Progressive headache 2 months | Right inferior fontal gyrus, CST was compressed and infiltrated | MMT 4 | grade 2 |
| 30 | F | 27 | Giant cell glioblastoma(WHO Ⅳ) | Sudden twitch one day | Right inferior fontal gyrus, CST was compressed and infiltrated | MMT 4 | grade 2 |
| 31 | F | 43 | Glioblastoma(WHO Ⅳ) | Headache and dizziness for half a month | Left frontal and temporal junction areas, CST was compressed | MMT 4 | grade 1 |
| 32 | M | 41 | Glioblastoma(WHO Ⅳ) | Intermittent headache, vomiting with recurrent seizures 20 days | Left middle frontal gyrus, CST was compressed | MMT 4 | grade 1 |
| 33 | M | 16 | Glioblastoma(WHO Ⅳ) | Have a headache for 1 years | Left temporal lobes, CST was compressed | MMT 4 | grade 1 |
| 34 | M | 45 | Anaplastic astrocytoma(WHO Ⅲ) | Headache, anorexia, muscle strength decreased | Left temporal lobes, CST was compressed and infiltrated | MMT 4 | grade 2 |
| 35 | F | 48 | Anaplastic astrocytoma(WHO Ⅲ) | Right limb muscle strength decreased, right facial numbness | Left inferior fontal gyrus, CST was compressed | MMT 4 | grade 1 |
| 36 | M | 24 | Anaplastic astrocytoma(WHO Ⅲ) | Limb numbness | Left middle frontal gyrus, CST was compressed and infiltrated | MMT 4 | grade 2 |
| 37 | M | 8 | Anaplastic astrocytoma(WHO Ⅲ) | Headache dizziness 20 days | Right middle fontal gyrus, CST was compressed | MMT 4 | grade 1 |
| 38 | M | 41 | Glioblastoma(WHO Ⅳ) | The causes of convulsion of unknown origin | Left postcentral gyrus, CST was compressed | MMT 4 | grade 1 |
| 39 | M | 40 | Glioblastoma(WHO Ⅳ) | Limb weakness | Left parietal cortex, CST was compressed | MMT 4 | grade 1 |
| 40 | F | 40 | Glioblastoma(WHO Ⅳ) | Dizziness 6 months | Left inferior frontal lobe, CST was compressed | MMT 5 | grade 1 |
| 41 | F | 44 | Astrocytoma(WHO Ⅱ) | Dizziness, headache six months | Left frontal and temporal junction areas, CST was compressed | MMT 5 | grade 1 |
| 42 | M | 43 | Anaplastic oligodendroglioma(WHO Ⅲ) | Repeated headache for 4 months, increased 10 days | Left middle frontal gyrus, CST was compressed | MMT 5 | grade 1 |
| 43 | F | 62 | Oligodendroglioma(WHO Ⅱ) | Dizziness 2 days | Left temporal lobes, CST was compressed | MMT 5 | grade 1 |
| 44 | F | 48 | Astrocytoma(WHO Ⅱ) | Intermittent headache for 1 months | Left middle frontal gyrus, CST was compressed | MMT 5 | grade 1 |
| 45 | M | 29 | Astrocytoma(WHOⅠ) | Dizziness 3 months | Left frontal and temporal junction areas, CST was compressed | MMT 5 | grade 1 |
