# Supplementary material for: A pilot study of pre-operative motor dysfunction from gliomas in the region of corticospinal tract: Evaluation with diffusion tensor imaging
Source: PLoS One. 2017 Aug 22;12(8):e0182795. doi: 10.1371/journal.pone.0182795 (PMC5568729; doi:10.1371/journal.pone.0182795)
Supplement: S2 Table — Note: rFA was the injuryed/contralateral FA value of injured and contralateral side in posterior limb of internal capsule, rFDi was the injuryed/contralateral FDi value of injured and contralateral CST, test level to P <0.05 was considered statistically significant, * indicated statistically significant differences. (DOCX) [file pone.0182795.s004.docx]

**Table 2. The comparison of rFDi and rFA among different** **muscle groups**

| comparison among muscle strength groups | rFA | | rFDi | |
| --- | --- | --- | --- | --- |
|  | t value | *P* value | t value | *P* value |
| 2 and 3 | -8.22 | <0.001^＊^ | -3.29 | 0.091 |
| 2 and 4 | -8.90 | <0.001^＊^ | -3.10 | <0.001^＊^ |
| 2 and 5 | -12.02 | <0.001^＊^ | -6.94 | 0.001^＊^ |
| 3 and 4 | -1.25 | 0.775 | -2.25 | 0.016^＊^ |
| 3 and 5 | -5.10 | 0.002^＊^ | -5.91 | 0.018^＊^ |
| 4 and 5 | -2.99 | 0.041^＊^ | 0.59 | 0.481 |

Note: rFA was the injuryed/contralateral FA value of injured and contralateral side in posterior limb of internal capsule, rFDi was the injuryed FDi/contralateral Fdi value of injured and contralateral CST, test level to P <0.05 was considered statistically significant, * indicated statistically significant differences.
