# Supplementary material for: A pilot study of pre-operative motor dysfunction from gliomas in the region of corticospinal tract: Evaluation with diffusion tensor imaging
Source: PLoS One. 2017 Aug 22;12(8):e0182795. doi: 10.1371/journal.pone.0182795 (PMC5568729; doi:10.1371/journal.pone.0182795)
Supplement: S1 File — (DOCX) [file pone.0182795.s005.docx]

STROBE Statement—checklist of items that should be included in reports of observational studies

|  | Item No. | Recommendation | Page  No. | Relevant text from manuscript |
| --- | --- | --- | --- | --- |
| **Title and abstract** | 1 | (*a*) Indicate the study’s design with a commonly used term in the title or the abstract | 1 | Brain tumors in the corticospinal tract (CST) region are more likely to cause motor dysfunction. |
|  |  | (*b*) Provide in the abstract an informative and balanced summary of what was done and what was found | 1 | We applicate DTI to evaluate the effect of gliomas located in the CST region on motor function |
| Introduction | | | |  |
| Background/rationale | 2 | Explain the scientific background and rationale for the investigation being reported | 1-2 | Brain tumors occurring in the motor cortex may cause reorganization of the motor function area^[1]^. Therefore, tumors in these regions may not necessarily cause severe limb dysfunction. |
| Objectives | 3 | State specific objectives, including any prespecified hypotheses | 2 | We utilized DTI to study the effect of glioma located in the CST region on motor function prior to surgical intervention. |
| Methods | | | |  |
| Study design | 4 | Present key elements of study design early in the paper | 3 | Supratentorial gliomas at Guizhou Medical University Hospital and Enshi Central Hospital were enrolled in this study from February 2013 to April 2016 |
| Setting | 5 | Describe the setting, locations, and relevant dates, including periods of recruitment, exposure, follow-up, and data collection |  | The posterior limb of the internal capsule (FA) and relative FA were measured. Injury of CST from tumor was divided into three grades. The fiber density index (FDi) and relative FDi of injured and contralateral CST were measured. The correlations between muscle strength and the CST injury grade and the rFA, affected FDi, rFDi values were calculated |
| Participants | 6 | (*a*) *Cohort study*—Give the eligibility criteria, and the sources and methods of selection of participants. Describe methods of follow-up  *Case-control study*—Give the eligibility criteria, and the sources and methods of case ascertainment and control selection. Give the rationale for the choice of cases and controls  *Cross-sectional study*—Give the eligibility criteria, and the sources and methods of selection of participants | 3-6 | **Case collection and scanning methods:**Tumors were diagnosed with pre-operative conventional and contrast-enhanced MRI and were classified according to World Health Organization 2007 criteria as confirmed by surgery and biopsy.This study identified the inclusion and exclusion criteria.  **MR sacn:** All patients underwent conventional contrast-enhanced brain MRI and DTI scan protocols.  **Measurement of Relevant parameters:** A region of interest (ROI) area measuring about 50 mm^2^ was placed in the posterior three quarters of the posterior limb of the internal capsule of the affected side by two experienced neuroradiologists who had at least five years of work experience and were familiar with the application of the workstation. Seed regions were selected using the axial color encoding direction map for CST fiber tracking. |
|  |  | (*b*)*Cohort study*—For matched studies, give matching criteria and number of exposed and unexposed  *Case-control study*—For matched studies, give matching criteria and the number of controls per case |  |  |
| Variables | 7 | Clearly define all outcomes, exposures, predictors, potential confounders, and effect modifiers. Give diagnostic criteria, if applicable | 6 | Contralateral knee muscle strength was tested in all patients using the manual muscle testing (MTT) The knee muscle strength was divided into 0-5 Grade (from 0: no movement to 5: normal movement), Injury to the CST from glioma was divided into three grades (grade 1: displacement, grade 2: infiltration, grade 3: disruption). |
| Data sources/measurement | 8* | For each variable of interest, give sources of data and details of methods of assessment (measurement). Describe comparability of assessment methods if there is more than one group | *5* | Seed regions were selected using the axial color encoding direction map for CST fiber tracking. A line propagation technique was used to track fibers and the logic algorithm "AND" was used to include the three ROIs through the fiber tracts, as well as "NOT" to exclude fiber tracts deviating from normal anatomy. Using the contralateral corresponding region as a reference, ipsilateral and contralateral CST FDI and rFDi were obtained. The FA threshold was set to 0.2, the angle of fiber tracing was 27 degrees, and step size was 0.2. |
| Bias | 9 | Describe any efforts to address potential sources of bias | 5 | FA values were measured by two independent readers on the axial FA color-coded map of the healthy and injured side. ROI measurement of the healthy and the injured side were repeated three times and an average value was recorded. This value was used to calculate relative FA values (rFA = injured FA/contralateral FA). |
| Study size | 10 | Explain how the study size was arrived at | 3 | Fifty-two patients with suspected primary supratentorial gliomas at Guizhou Medical University Hospital and Enshi Central Hospital were enrolled in this study from February 2013 to April 2016. |

Continued on next page

| Quantitative variables | 11 | Explain how quantitative variables were handled in the analyses. If applicable, describe which groupings were chosen and why | 6 | **All gliomas were classified as low grade and high grade,** because the high level and low grade tumors had large differences in biological behavior.  **Classify the status of the CST relative to tumors.** Because nearly all patients in this study had displacement of the CST to a certain extent, infiltration and disruption were classified separately: 1) grade 1: displacement - only white matter tracts signal normal on FA map or FA color-coded map; 2) grade 2: displacement and infiltration - displacement and abnormal signal on FA map or FA color-coded map with decreased FA values; white matter fiber tracts surrounded by tissue edema but remained intact without significant disruption ; 3) grade 3: displacement and disruption - coexistence of white matter fiber tracts displacement and disruption; the direction of white matter tracts cannot be identified on FA map or FA color-coded map  **Contralateral knee muscle strength** was tested in all patients using the manual muscle testing (MTT) according to the Lovett staging classification (Robert Lovett taxonomy). The knee muscle strength was divided into 0-5 Grade (from 0: no movement to 5: normal movement) |
| --- | --- | --- | --- | --- |
| Statistical methods | 12 | (*a*) Describe all statistical methods, including those used to control for confounding | 6 | All statistical analysis was performed using SPSS 13.0  The differences between the mean rFA and rFDi values of different muscle strength groups were analyzed with analysis of variance (ANOVA). The difference of muscle strength between low and high grade gliomas groups were analysed with Mann-Whitney U-test. |
|  |  | (*b*) Describe any methods used to examine subgroups and interactions | 6 | The rFA and rFDi values of each muscle strength group were tested for normality as well as equality of variances. |
|  |  | (*c*) Explain how missing data were addressed |  | This section excludes this study |
|  |  | (*d*) *Cohort study*—If applicable, explain how loss to follow-up was addressed  *Case-control study*—If applicable, explain how matching of cases and controls was addressed  *Cross-sectional study*—If applicable, describe analytical methods taking account of sampling strategy |  | According to the grade of CST injury groups, muscle strength groups with the DTI parameters to analysis. |
|  |  | (*e*) Describe any sensitivity analyses | 6 | Statistical significance was designated as P < 0.05. |
| Results | | | | |
| Participants | 13* | (a) Report numbers of individuals at each stage of study—eg numbers potentially eligible, examined for eligibility, confirmed eligible, included in the study, completing follow-up, and analysed | 7 | Of our 52 patients, 4 cases with glioma partial infiltration in the primary motor cortex and 3 cases with very large tumors that precluded fiber tracking were excluded. |
|  |  | (b) Give reasons for non-participation at each stage |  | In the exclusion of the standard range |
|  |  | (c) Consider use of a flow diagram |  | Only study design using flow chart |
| Descriptive data | 14* | (a) Give characteristics of study participants (eg demographic, clinical, social) and information on exposures and potential confounders | 7 | Thirty-eight cases demonstrated motor dysfunction. Seven cases exhibited headache, dizziness, convulsions and slight movement dysfunction. |
|  |  | (b) Indicate number of participants with missing data for each variable of interest |  | 7 cases |
|  |  | (c) *Cohort study*—Summarise follow-up time (eg, average and total amount) |  | About one week after surgery |
| Outcome data | 15* | *Cohort study*—Report numbers of outcome events or summary measures over time | *3* | 3 years |
|  |  | *Case-control study—*Report numbers in each exposure category, or summary measures of exposure |  |  |
|  |  | *Cross-sectional study—*Report numbers of outcome events or summary measures |  |  |
| Main results | 16 | (*a*) Give unadjusted estimates and, if applicable, confounder-adjusted estimates and their precision (eg, 95% confidence interval). Make clear which confounders were adjusted for and why they were included |  | Tumor compression may lead to ROI slightly different |
|  |  | (*b*) Report category boundaries when continuous variables were categorized | 7 | we enrolled 45 cases that satisfied our inclusion criteria. The average age was 45.3 (range 6-78) years. Twenty-six were male and 19 were female. |
|  |  | (*c*) If relevant, consider translating estimates of relative risk into absolute risk for a meaningful time period | 7 | A negative correlation was found between muscle strength and CST injury grade (r = -0.840, *P* < 0.05) while a positive correlation was found between muscle strength and rFA value of the posterior limb of internal capsule between the injured and contralateral side (r = 0.615, *P* < 0.05).  **The relative risk** is that the sample size is not large enough |

Continued on next page

| Other analyses | 17 | Report other analyses done—eg analyses of subgroups and interactions, and sensitivity analyses | 6 | The relationship between injury degrees of CST, rFA, FDi, rFDi values and muscle strength were analyzed with Spearman rank correlation analysis.  Statistical significance was designated as P < 0.05. |
| --- | --- | --- | --- | --- |
| Discussion | | | | |
| Key results | 18 | Summarise key results with reference to study objectives | 7 | A negative correlation was found between muscle strength and CST injury grade while a positive correlation was found between muscle strength and rFA value between the injured and contralateral side. |
| Limitations | 19 | Discuss limitations of the study, taking into account sources of potential bias or imprecision. Discuss both direction and magnitude of any potential bias | 10 | Linear extension technology tracing of fiber tracts is based on the diffusion property of water molecules in each voxel, not true fiber tracts.  Our results were not confirmed by histopathology  Only knee muscle strength was tested, but tumors can involve any portion of the CST resulting in heterogeneous distribution of upper and lower limb weakness. |
| Interpretation | 20 | Give a cautious overall interpretation of results considering objectives, limitations, multiplicity of analyses, results from similar studies, and other relevant evidence | 8-9 | DTI parameters can potentially quantify the degree of CST injury and assess the damage to motor conduction fibers preoperatively, and also can predict therapeutic effect and outcome.  Changes of muscle strength due to CST injury may be the result of tumor cell proliferation and infiltration degree. |
| Generalisability | 21 | Discuss the generalisability (external validity) of the study results | 11-12 | Our study suggests that pre-operative DTI quantitative parameters can assess the degree of CST injury by glioma. |
| Other information | |  | | |
| Funding | 22 | Give the source of funding and the role of the funders for the present study and, if applicable, for the original study on which the present article is based |  | Funding: Bo Gao—RELATED: Grant: The imaging genomic mapping of multiple mode MR in gliomas Grant # ZR2014HL084. *MSS Mark S. Shiroishi* was partially supported by SC CTSI (NIH/NCRR/NCATS) Grant # KL2TR000131. |

*Give information separately for cases and controls in case-control studies and, if applicable, for exposed and unexposed groups in cohort and cross-sectional studies.

**Note:** An Explanation and Elaboration article discusses each checklist item and gives methodological background and published examples of transparent reporting. The STROBE checklist is best used in conjunction with this article (freely available on the Web sites of PLoS Medicine at http://www.plosmedicine.org/, Annals of Internal Medicine at http://www.annals.org/, and Epidemiology at http://www.epidem.com/). Information on the STROBE Initiative is available at www.strobe-statement.org.
